# Clinical utility of tumor genomic profiling in patients with high plasma circulating tumor DNA burden or metabolically active tumors

**DOI:** 10.1186/s13045-018-0671-8

**Published:** 2018-11-06

**Authors:** Cathy Zhou, Zilong Yuan, Weijie Ma, Lihong Qi, Angelique Mahavongtrakul, Ying Li, Hong Li, Jay Gong, Reggie R. Fan, Jin Li, Michael Molmen, Travis A. Clark, Dean Pavlick, Garrett M. Frampton, Brady Forcier, Elizabeth H. Moore, David K. Shelton, Matthew Cooke, Siraj M. Ali, Vincent A. Miller, Jeffrey P. Gregg, Philip J. Stephens, Tianhong Li

**Affiliations:** 1 Department of Radiology, University of California Davis School of Medicine, Sacramento, CA USA; 2 University of California Davis Comprehensive Cancer Center, Sacramento, CA USA; 3 Department of Radiology, Hubei Cancer Hospital, Tongji Medical College, Huazhong University of Science and Technology, Wuhan, China; 4 Division of Hematology and Oncology, Department of Internal Medicine University of California Davis School of Medicine, 4501 X Street, Suite 3016, Sacramento, CA 95817 USA; 5 Department of Public Health Sciences, University of California, Davis, CA USA; 6 Currently Department of Medical Oncology, Chinese PLA General Hospital, Beijing, China; 7 Currently Department of Geriatrics, Peking University First Hospital, Beijing, China; 8 Foundation Medicine, Inc., Cambridge, MA USA; 9 Department of Pathology and Laboratory Medicine and Genomic Shared Resource, University of California Davis School of Medicine, Sacramento, CA USA; 10 Department of Internal Medicine, Veterans Affairs Northern California Health Care System, Mather, CA USA

**Keywords:** Next-generation sequencing (NGS), Plasma, Cell-free DNA (cfDNA), Circulating tumor DNA (ctDNA), Genomic alterations (GAs), Maximum somatic allele frequency (MSAF), Positron emission tomography (PET) scan, Maximum standardized uptake value (SUVmax)

## Abstract

**Background:**

This retrospective study was undertaken to determine if the plasma circulating tumor DNA (ctDNA) level and tumor biological features in patients with advanced solid tumors affected the detection of genomic alterations (GAs) by a plasma ctDNA assay.

**Method:**

Cell-free DNA (cfDNA) extracted from frozen plasma (*N* = 35) or fresh whole blood (*N* = 90) samples were subjected to a 62-gene hybrid capture-based next-generation sequencing assay FoundationACT. Concordance was analyzed for 51 matched FoundationACT and FoundationOne (tissue) cases. The maximum somatic allele frequency (MSAF) was used to estimate the amount of tumor fraction of cfDNA in each sample. The detection of GAs was correlated with the amount of cfDNA, MSAF, total tumor anatomic burden (dimensional sum), and total tumor metabolic burden (SUVmax sum) of the largest ten tumor lesions on PET/CT scans.

**Results:**

FoundationACT detected GAs in 69 of 81 (85%) cases with MSAF > 0. Forty-two of 51 (82%) cases had ≥ 1 concordance GAs matched with FoundationOne, and 22 (52%) matched to the National Comprehensive Cancer Network (NCCN)-recommended molecular targets. FoundationACT also detected 8 unique molecular targets, which changed the therapy in 7 (88%) patients who did not have tumor rebiopsy or sufficient tumor DNA for genomic profiling assay. In all samples (*N* = 81), GAs were detected in plasma cfDNA from cancer patients with high MSAF quantity (*P* = 0.0006) or high tumor metabolic burden (*P* = 0.0006) regardless of cfDNA quantity (*P* = 0.2362).

**Conclusion:**

This study supports the utility of using plasma-based genomic assays in cancer patients with high plasma MSAF level or high tumor metabolic burden.

## Background

The clinical application of multiplexed molecular biomarker assays has revolutionized cancer diagnosis and treatment, enabling the current era of precision cancer medicine [1–4]. Historically, the majority of current gold-standard biomarker assays were developed with archival tumor specimens obtained via invasive biopsies or surgical procedures [1, 5, 6]. Unfortunately, these assays can fail due to insufficient tumor specimen acquisition in up to 30% of reported cases [7–9]. Furthermore, biopsy may not be feasible in patients who are critically ill, whose tumors are in inaccessible locations, or need serial biopsies. Liquid biopsy and genomic profiling assays of plasma circulating tumor DNA (ctDNA) have been recommended by the College of American Pathologists (CAP), the International Association for the Study of Lung Cancer (IASLC), and the Association for Molecular Pathology (AMP) guidelines and are increasingly used in the clinic when tumor tissue is limited and/or insufficient for molecular testing [10–13]. Plasma ctDNA is defined as tumor-derived fragmented DNA in the blood that is not associated with cells. Plasma ctDNA should not be confused with cell-free DNA (cfDNA), which refers to DNA that is freely circulating in the bloodstream from both tumor and non-tumor origin. The detection of plasma cfDNA and/or ctDNA in cancer patients has been correlated with high tumor stage [14, 15], metastasis [14], poor prognosis [16, 17], treatment response [18, 19], and recurrence [20, 21]. Alterations in the amount of ctDNA can reflect the dynamic changes of tumor metabolic burden during the disease course [22, 23]. Liquid biopsy has the advantages of being less or non-invasive with shorter turnaround time and less cost, while providing a temporal measurement of tumor burden and more fully capturing the landscape of tumor heterogeneity. However, the concordance rate of detected GAs between tissue and blood tumor genomic profiling assays varies significantly in reported studies [24–27]. While comparisons have been mostly focused on different analytic platforms, little attention has been paid to the unique tumor components needed for tissue and plasma tumor DNA. The sensitivity of detecting GAs and mutation allele frequencies (MAFs) in archived tissue is directly related to the absolute number and proportion of tumor cells present and extracted in the studied specimens [28]. Currently, plasma cfDNA from cancer patients, which includes both ctDNA and DNA from normal cells, has been used as a tumor resource in clinical assays for genomic profiling [29, 30]. The sensitivity of detecting GAs in plasma samples is presumably affected by the steady-state level of plasma ctDNA shed by viable tumor cells into the blood, its metabolism in the plasma, and its percentage in relation to the total amount of plasma cfDNA. cfDNA levels are quantified by polymerase chain reaction (PCR) correlated with SUVmax in patients with non-small-cell lung cancer (NSCLC) [31]. Little is known regarding the quantitative relationship between the relative and absolute level of ctDNA in the blood and the biological features of a tumor [32] and the impact of tumor metabolic burden on the successful detection of GAs using a NGS assay for plasma ctDNA in cancer patients [31, 33].

Positron emission tomography with computed tomography (PET/CT) has been routinely used as a noninvasive imaging tool for tumor staging, treatment planning, and treatment evaluation [34–36]. PET/CT scans provide a functional assessment of cancer cells based on increased glucose uptake and glycolysis, which may even detect metabolic abnormalities before morphologic or anatomic alterations occur in tumors [37, 38]. The standardized uptake value (SUV) is a semi-quantitative measurement of the tissue fluoro-D-glucose (FDG) accumulation rate, and the maximum standardized uptake value (SUVmax) has been used in the routine clinical report as the most reproducible imaging biomarker that has diagnostic and prognostic value on PET/CT scan [39, 40]. Decreased FDG activity has been correlated with the decreased plasma ctDNA level and tumor response in only a few prior cases [23, 41]. We hypothesized that the detection of GAs in plasma ctDNA depends on the presence of a sufficient amount of ctDNA produced by metabolically viable tumors in patients with advanced solid tumors. The objective of this study was to determine if the detection of GAs using the FoundationACT assay is associated with the amount of plasma cfDNA, plasma ctDNA, total tumor anatomic burden, or total tumor metabolic burden in cancer patients.

## Methods

### Patient population

**Fig. 1:**
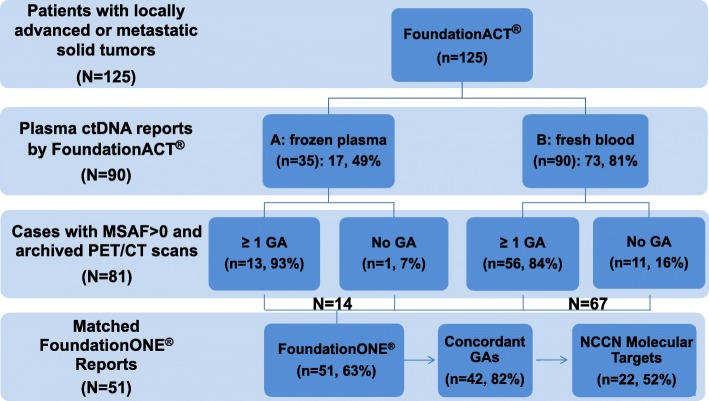
Study schema. This study included 125 consecutive patients with locally advanced or metastatic solid tumors who underwent FoundationACT testing between November 17, 2015, and April 17, 2017. Among 90 patients with available FoundationACT reports, 81 patients had no interval treatment and ^18^F-FDG PET/CT scans performed at our institution within 45 days of blood sampling for imaging quantification analyses. The concordance rate of GA detection between 51 matched FoundationACT and FoundationOne cases was calculated and further analyzed for NCCN-recommended molecular targets in 42 patients

Figure 1 shows the study flow chart. This institutional review board (IRB) approved study (#937274 at the University of California, Davis) retrospectively reviewed 125 consecutive patients with locally advanced or metastatic solid tumors who underwent FoundationACT testing between November 17, 2015, and April 17, 2017. Among 90 patients with available FoundationACT reports, 81 patients had detectable ctDNA as measured by MSAF > 0 in this study and quantitative PET/CT scans that were performed at our institution within 45 days of blood sampling. Furthermore, 51 of these 81 (63%) patients with no treatment between the tissue and liquid biopsies also had matched FoundationOne reports. The concordance GAs across the tissue and plasma assays was analyzed for all actionable molecular targets and the National Comprehensive Cancer Network (NCCN)-recommended molecular targets. The concordance rate of GAs detected between 51 matched FoundationACT and FoundationOne cases was calculated by dividing the number of concordant alterations by all the alterations (i.e., the concordant alterations plus unique alterations) detected by the FoundationOne assay. Only substitutions, insertion/deletions, and rearrangements were included; amplifications were not included.

### Preparation and quantification of plasma cell-free DNA

Twenty milliliters (mLs) of peripheral whole blood were collected in K3 EDTA tubes for frozen samples (cohort A) or in K3 EDTA-containing Streck tubes (Cell-Free DNA BCT) for fresh blood (cohort B). Circulating cfDNA was recovered from ~ 3 mL of frozen plasma (cohort A) or 4 to 5 mL of fresh plasma samples (cohort B) using the QIAamp Circulating Nucleic Acid Kit (Qiagen) and quantified using the Qubit 2.0 Fluorometer with dsDNA HS assay kits (Life Technologies, Carlsbad, CA). The tumor fraction of plasma cfDNA in each sample was estimated using the maximum somatic allele frequency (MSAF). MSAF is determined by calculating the allele fraction for all known somatic, likely somatic, and variant of unknown significance (VUS) alterations, excluding those alterations that are likely germline. The reported MSAF value for the clinical case was the highest allele frequency of the detected somatic variants. The logic utilizes both in-house and external references for germline information, including the Exome Aggregation Consortium (ExAC) database [12] and the Single Nucleotide Polymorphism Database (dbSNP) [42], as well as aneuploidy in the specimen’s copy number profile to better assess MSAF. Importantly, each variant was subject to manual curation by a team of expert genomic analysts to remove sequence or alignment artifacts.

### Analytic validation of the FoundationACT ctDNA assay

All samples with at least 20 ng cfDNA (20–100 ng) were subjected to the hybrid capture-based NGS (FoundationACT) assay performed at a Clinical Laboratory Improvement Amendments (CLIA)-certified, College of American Pathologists (CAP)-accredited, New York State-approved laboratory (Foundation Medicine, Inc). This assay identified four classes of genomic alterations: base substitutions, small insertions and deletions (indels), copy number variations, and rearrangements/fusions. The target region includes a total of 62 genes, with 61 genes sequenced across all exons and 6 genes across introns commonly involved in rearrangements [11, 43]. The FoundationACT assay has a sensitivity of ≥ 99.3% and a positive predictive value (PPV) of 100% for base substitutions at allele frequency (AF) > 0.4%, a sensitivity of ≥ 98.5%, and a PPV of > 100% for insertion/deletions and a sensitivity of > 99% and a PPV of 98.0% for rearrangements at AF > 1.0% [29]. Concordance rate was calculated by comparing GAs present in both FoundationACT and FoundationOne. Table 1 summarizes the genes tested in each assay.

**Table 1:**
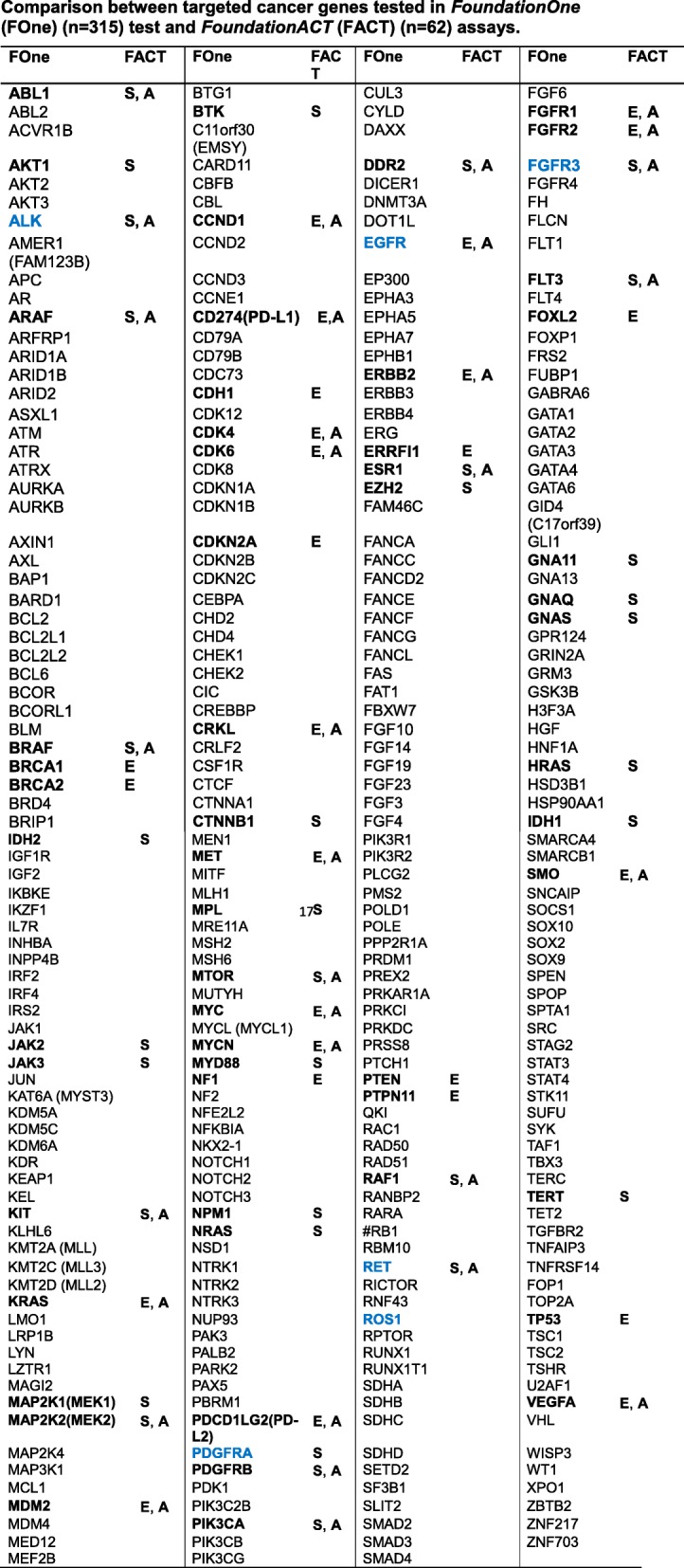
Comparison between targeted cancer genes tested in *FoundationOne* (FOne) (*n* = 315) test and *FoundationACT* (FACT) (*n* = 62) assays

Bold highlights the genes that are tested in both FoundationACT and FoundationOne. *E*, genes with entire coding sequence coverage (*n* = 27); *S*, genes with selected, critical exon coverage. Blue highlights the rearranged genes tested in both FoundationACT and FoundationOne assays (*n* = 6). *A*, genes that were evaluable for copy number amplification

### PET/CT data acquisition and image analysis

All PET/CT studies were performed on a Discovery 690 scanner (General Electric Company). After fasting for at least 6 h, patients with glucose levels below 200 mg/dL were administered 10 mCi of 2-deoxy-2-[fluorine-18]fluoro-D-glucose (^18^F-FDG) and allowed to rest quietly for a period of 60 min. Patients were then scanned from the head to mid-thigh, followed by 3D PET emission data collection, reconstructed using an ordered subset expectation maximization algorithm (2 subsets, 24 iterations) and CT-based attenuation correction. At least two readers who were blinded to the clinical and genomic data reviewed and verified the tumor lesions on each clinical report. While Response Evaluation Criteria in Solid Tumors (RECIST) version 1.1 limits measurable lesions to ≤ 2 per organ and ≤ 5 lesions in total to evaluate treatment response [44], we measured the dimension and metabolic activity of up to ten of the most prominent primary and metastatic lesions. The total tumor anatomic burden was defined as the sum of the lesion diameters, and the tumor metabolic activity was estimated by the sum of the SUVmax values.

### Statistical analysis

All the known and likely somatic alterations, substitutions, insertions/deletions, and rearrangements identified (designated as genomic alterations, GAs) in FoundationACT were used for the analysis. All data were summarized as the mean ± standard deviation (SD). Descriptive statistics for continuous and categorical variables were stratified by GA status. The two-sample *t* test was used for continuous variables. All analyses were conducted using SAS, university edition 2.5 9.4 M4 (SAS Institute, Cary, NC), and figures were made using GraphPad Prism software (Version 7.03). All statistical tests were two-sided and a *P* value less than 0.05 was considered statistically significant.

## Results

### Patient characteristics

Patient demographics and disease characteristics are summarized in Table 2. Of the entire study population (*N* = 81), the median age was 67 years (range 44–93) with 55 (68%) females. Fifty-three (63%) patients were Caucasian, 15 (19%) were Hispanic, 8 (10%) were Asian, and 7 (9%) were African American. Cancer types included predominantly lung adenocarcinoma (LUAD, *n* = 42, 52%), lung squamous cell carcinoma (LUSC, *n* = 17, 21%), and breast cancer (*n* = 16, 20%).

**Table 2:** Patient demographics and sample information

	Group A (*N* = 14)	Group B (*N* = 67)	Total (*N* = 81)
Type of specimen	Frozen plasma	Fresh whole blood	All samples
Age: median (range)	68 (44–73)	67 (45–93)	67 (44–93)
Gender: female *N* (%)	9 (64%)	46 (69%)	55 (68%)
Race/ethnicity: *N* (%)			
Caucasian	8 (57%)	43 (64%)	51 (63%)
Asian	2 (14%)	13 (19%)	15 (19%)
Hispanic	2 (14%)	6 (9%)	8 (10%)
African American	2 (14%)	5 (7%)	7 (9%)
Cancer type/histology: *N* (%)			
LUAD	2 (14%)	40 (60%)	42 (52%)
LUSC	9 (64%)	8 (12%)	17 (21%)
Breast	3 (21%)	13 (19%)	16 (20%)
Other cancer types*	0	6 (9%)	6 (7%)

*Other cancer types include lung small cell carcinoma (*n* = 2); lung large cell neuroendocrine (*n* = 1); ovarian, adenocarcinoma (*n* = 2); prostate, adenocarcinoma (*n* = 1)

*LUAD* lung adenocarcinoma, *LUSC* lung squamous cell carcinoma

### Detection of GAs in plasma ctDNA

The yield of plasma cfDNA was highly variable among cancer patients and was significantly higher in fresh blood group compared to frozen plasma group (14.2 ± 32.2 vs 2.5 ± 5.0 ng/mL, *P* = 0.023) (Table 3). Of note, lower volumes of plasma were used in the frozen plasma group vs fresh blood group (3.0 vs 8.5 ml). A total of 215 GAs were detected in 81 patients, with an average of 2.7 GAs per sample (range 1–11), including 154 (72%) base substitutions, 34 (16%) insertions/deletions, 14 (7%) amplifications, and 13 (6%) rearrangements/fusions (Table 3).

**Table 3:** GAs and factors affect the detection of GAs in the plasma ctDNA assay

FoundationACT	Group A (*N* = 14)	Group B (*N* = 67)	Total (*N* = 81)
Specimen type	Frozen plasma	Fresh whole blood	All samples
Volume (mL)	~ 3.0	8.5 (5.5–11.5)	8.0 (3.0–11.5)
cfDNA (ng/mL)	2.5 ± 5.0	14.2 ± 32.2	*P* = 0.023
GA ≥ 1	13 (93%)	56 (84%)	69 (85%)
Total number of GAs (average/case, range)	49 (3.5/case; 1–9)	166 (2.5/case; 1–11)	215 (2.7/case; 1–11)
Base substitutions	36 (73%)	118 (71%)	154 (72%)
Insertions/deletions	8 (16%)	26 (16%)	34 (16%)
Amplifications	1 (2%)	13 (8%)	14 (7%)
Rearrangements/fusions	4 (8%)	9 (5%)	13 (6%)
cfDNA (mean ± SD) ng/mL; (GA ≥ 1 vs GA = 0)	3.1 ± 5.9 vs 0.3 ± 0.1 (*P* = 0.5293)	8.6 ± 18.1 vs 18.9 ± 51.6 (*P* = 0.2314)	7.6 ± 16.7 vs 16.3 ± 47.3 (*P* = 0.2362)
MSAF (mean ± SD); (GA ≥1 vs GA = 0)	0.0648 ± 0.0823 vs 0.0005 ± 0.0007 (*P* = 0.3062)	0.1219 ± 0.2031 vs 0.0003 ± 0.0009 (*P* = 0.0432)	0.1117 ± 0.1880 vs 0.0003 ± 0.0009 (*P* = 0.0304)
Tumor burden by RECIST V1.1 cm (GA ≥ 1 vs GA = 0) (mean ± SD)	7.6 ± 6.4 vs 12.0 ± 12.0 (*P* = 0.4610)	9.8 ± 5.8 vs 5.7 ± 3.4 (*P* = 0.0292)	9.4 ± 5.9 vs 6.6 ± 5.3 (*P* = 0.1301)
Tumor metabolic activity by SUVmax mg/dL (GA ≥1 vs GA = 0) (mean ± SD)	51.1 ± 34.8 vs 31.2 ± 30.7 (*P* = 0.4772)	48.1 ± 29.5 vs 14.3 ± 12.6 (*P* = 0.0006)	48.6 ± 30.2 vs 16.9 ± 15.8 (*P* = 0.0006)

*GAs* genomic alterations, *cfDNA* cell-free DNA, *MSAF* maximum somatic allele frequency, *SUVmax* maximum standardized uptake value

**Fig. 2:**
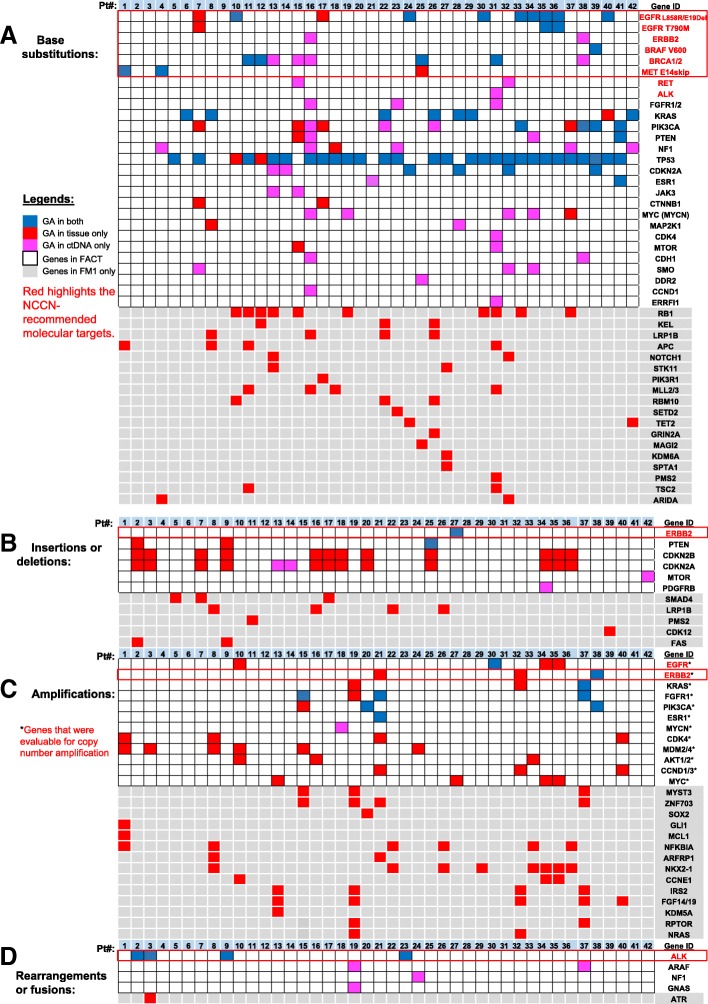
Genomic alterations (GAs) and concordance of NCCN-recommended molecular targets detected by both FoundationACT and FoundationOne assays in 42 patients with advanced solid tumors. GAs in base substitutions (**A**), insertions or deletions (**B**), amplifications (**C**) or rearrangements (**D**) detected in patients with detectable ctDNA (i.e., MSAF > 0) are shown. Concordant/shared GAs are in blue, GA found only in tissue are in red, and GA found only in ctDNA are in pink. Genes that were only included in tissue (FoundationOne) were shaded in gray. Red color highlights the NCCN-recommended test genes, and red box highlights the NCCN-recommended molecular targets

FoundationACT detected GAs in 69 (77%) patients (*N* = 90). When only considering samples with MSAF > 0, FoundationACT detected GAs in 69 (85%) samples (*N* = 81): 13 (93%) in the frozen plasma group and 56 (84%) in the fresh blood group. Fifty-one of these 69 (74%) cases have matched FoundationOne reports (Fig. 1). Figure 2 provides the detail of each genomic alteration detected by FoundationOne only, FoundationACT only, or both. Similar to a recent report [45], there were more concordance GAs in base substitutions in TP53 (93%), KRAS (88%), EGFR (77%), and PIK3CA (50%) genes (Fig. 2a, blue) than genes in the insertions or deletions, amplifications, or rearrangements (Fig. 2b–d) in our dataset. FoundationOne (gray) detected more GAs (in red) as there were more genes tested in FoundationOne (gray shade) than FoundationACT. Of 51 patients with matched FoundationOne and FoundationACT reports, 42 (82%) patients had at least one concordance GAs, which included 20 (48%) patients with non-NCCN-recommended and 22 (52%) patients with NCCN-recommended (red boxes in Fig. 2) molecular targets. FoundationACT detected 8 unique and 22 concordant GAs, with an overall concordance of 81% (range 67%–100%) (Table 4). Of 8 unique GAs identified by FoundationACT only, the liquid biopsy genomic data led to a change in the clinical care with clinical benefit in 7 (88%) patients who could not have tumor rebiopsy or sufficient tumor DNA for genomic profiling assay.

**Table 4:** Concordance of NCCN-recommended molecular targets detected by both FoundationACT and FoundationOne assays in 42 patients with advanced solid tumors

Tumor types	Genomic alterations	No. concordance (*N* = 22)	No. unique to FoundationOne (*N* = 4)	% concordance (*N* = 26)	No. unique to FoundationACT (*N* = 8)
NSCLC (*N* = 33)	EGFR L858R and exon 19 deletions	8	2	80%	0
	EGFR T790 M	2	1	67%	0
	ALK rearrangements	4	0	100%	0
	BRAF V600 mutation	1	0	100%	0
	MET exon 14 skip site alterations	2	1	67%	0
	ERBB2 mutations	0	0	100%	2
Breast (*N* = 7)	ERBB2 amplification or mutation	1	0	100%	1
	BRCA1/2 mutations	2	0	100%	4
Ovarian (*N* = 2)	BRCA1/2 mutations	2	0	100%	0

*NCCN* National Comprehensive Cancer Network, *NSCLC* non-small-cell lung carcinoma

**Fig. 3:**
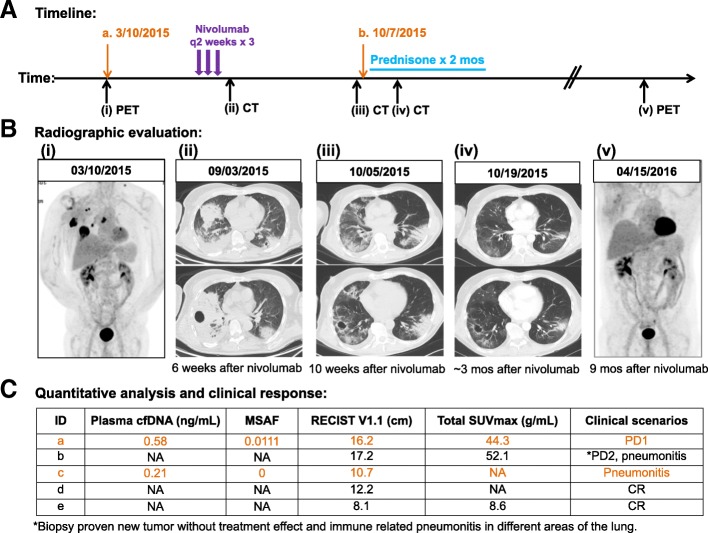
MSAF is a valid tool for quantifying the tumor fraction of cfDNA. A representative case showing MSAF was a better tool than cfDNA and SUVmax to correlate with clinical response when the patient had non-infectious, immune-related pneumonitis. **A** Schema of the clinical course. **B** Radiographic evaluation: a 67-year-old Caucasian male, former four pack-year smoking history (quit 20 years ago), presented with refractory, lung squamous cell carcinoma (**i**). The patient developed non-productive cough and shortness of breath after three doses of nivolumab monotherapy and was found to have biopsy-proven, new tumor formation in right lower lobe as well as grade 3 pneumonitis in bilateral lung fields (**ii**) [46]. However, notable tumor shrinkage at several pre-existing tumors was observed. Blood drawn at 10 weeks later before the initiation of high dose steroids revealed non-detectable ctDNA (i.e., MSAF zero) (**iii**). Despite discontinuation of nivolumab and use of steroids for over 2 months for symptomatic pneumonitis, continued tumor response to a complete remission was evident by radiographic assessment by ~ 3 months (**iv**), which has been maintained at 9 months (**v**) and a recent 3-year follow-up (*data not shown*). **C** Quantitative analysis of biomarkers and clinical responses were summarized in table

Twelve (15%) cases had no detected ctDNA (i.e., MSAF = 0) and no detectable GA. Table 5 summarizes the demographic and clinical information of these 12 cases, which included 6 (50%) were in complete remission (CR), 1 (8.3%) had partial response (PR), 3 (33%) had small volume, stable or indolent disease, and 2 (16.7%) had low volume progressive disease (PD). These data showed that cases with no ctDNA measured by MSAF correlated well with the relatively indolent disease. MSAF is particularly helpful to identify these cases with cfDNA mainly from non-cancerous sources, such as cancer patients who had a putative germline mutation (patient 7 and 8; both mutations were present in the corresponding tissue tumor DNA by FoundationOne), infection (patient 8 and 9), or non-infectious, immune-related pneumonitis (patient 6 and Fig. 3) [46]. After first blood draw (Fig. 3A(a)) and PET scan (Fig. 3B(i)), patient 6 received several cycles of chemotherapy and three doses of nivolumab before chest CT scan 6 months later (Fig. 3 B(ii). Although there was no treatment between CT scan (Fig. 3B(ii)) and blood collection (Fig. 3A(b)) and CT scan (Fig. 3 B(iii)) about 1 month apart, the activated, potent immune response in patient 6 led to complete remission (CR) of tumors by radiographic assessment at ~ 3 months (Fig. 3 B(iv)) at which time blood was collected. Figure 3 C summarizes the quantitative analysis of biomarkers and clinical response at each time point. Among these biomarkers, MSAF stands out as the most informative measurement of plasma tumor burden in the dynamic clinical scenarios for this patient. The patient remained in CR at 6 months (Fig. 3 B(v)) and 3-year clinical follow-up at the time of this submission (*data not shown*).

**Table 5:** Clinical scenarios of metastatic cancer patients with no detectable ctDNA (i.e., MSAF = 0)

Pt No.	Age (years old)	Gender	Race/ethnicity	Diagnosis	Cohort (A = frozen plasma; B=fresh blood)	cfDNA ng/mL	GA MAF (%)	MSAF	RECIST V1.1 (cm)	Total SUVmax	Clinical scenario
Pt 1	59	F	Not Hispanic, White	Breast, IDC	B	0.55	No reportable GA	0	1.8	N/A	CR
Pt 2	52	F	Not Hispanic, Asian	LUAD	B	4.20	No reportable GA	0	2.3	1.1	CR
Pt 3	68	M	Not Hispanic, White	LUAD	B	0.49	No reportable GA	0	2.5	2.6	CR
Pt 4	54	M	Not Hispanic, Filipino	LUAD	B	12.21	No reportable GA	0	6.2	5.8	CR
Pt 5	65	F	Not Hispanic, White	LUAD	B	0.75	No reportable GA	0	7.3	14.4	CR
Pt 6	68	M	Not Hispanic, White	LUSC	A	0.21	No reportable GA	0	20.5	52.9	*CR (ongoing at 3 years), pneumonitis
Pt 7	55	F	Not Hispanic, Asian	LUAD	B	2.94	TP53 E358V (49.1%)	0	2.3	4.3	PR
Pt 8	73	F	Not Hispanic, White	LUAD	B	7.25	BRCA2 S1121* (44.6%)	0	7.9	14.6	PD vs inflammation
Pt 9	68	F	Not Hispanic, White	LUAD	B	182.16	No reportable GA	0	5.8	34.1	PD vs inflammation
Pt 10	84	M	Not Hispanic, White	LUAD	B	9.04	No reportable GA	0	7.7	30.2	Small volume, indolent disease
Pt 11	67	F	Not Hispanic, Iran	Breast, inflammatory	B	5.93	No reportable GA	0	9.6	11.3	Small volume, indolent disease vs pneumonia
Pt 12	54	M	Not Hispanic, Filipino	LUAD	B	0.05	No reportable GA	0	10.8	32.0	Small volume, indolent disease vs pneumonia

*CR* complete response, *cfDNA* cell free DNA, *F* female, *GA* genomic alteration, *IDC* invasive ductal carcinoma, *LUAD* lung adenocarcinoma, *LUSC* lung squamous cell carcinoma, *M* male, *MAF* mutation allele frequency, *MSAF* maximum somatic allele frequency, *NA* not available, *PR* partial response, *Pt* patient, *RECIST* Response Evaluation Criteria in Solid Tumors, *SD* stable disease, *SUVmax* maximum standardized uptake value

*Biopsy proven new tumor without treatment effect and immune related pneumonitis in different areas of the lung

### Effect of tumor-specific factors on the detection of genomic alterations in plasma ctDNA

**Fig. 4:**
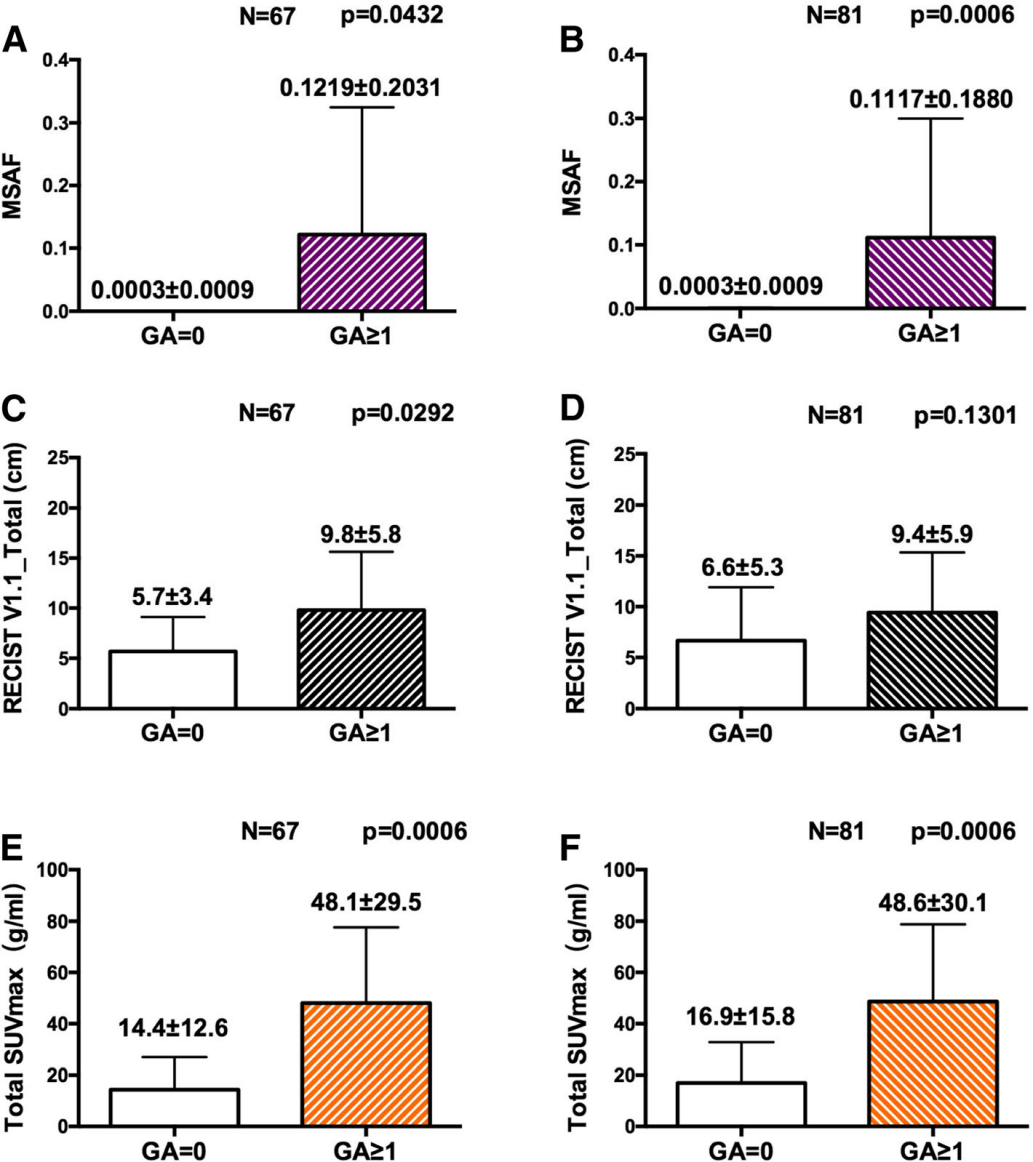
Factors affect the detection of GAs in FACT assay. Comparison of MSAF, tumor anatomic burden, and tumor metabolic burden in relation to the detection of GAs (0 vs ≥ 1) in patients with fresh blood samples (**a**, **c**, and **e**) and all sample group (**b**, **d*,*** and **f**). Each bar demonstrates a mean ± SD. *P* < 0.05 by two-sample *t* test is considered statistically significant

We next determined the impact of four tumor-specific factors on the ability to detect GAs by FoundationACT. The quantity of cfDNA did not affect the detection of GAs in both sample groups (Table 3). MSAF was significantly higher in GA ≥ 1 versus GA = 0 cohorts in the fresh blood specimens (Fig. 4a) and all samples (Fig. 4b). The amount of anatomic tumor burden by total RECIST V1.1 correlated with the detection of GAs in the fresh blood group (9.8 ± 5.8 vs 5.7 ± 3.4 cm, *P* = 0.0292; Fig. 4c) but not in the all sample group (9.4 ± 5.9 vs 6.6 ± 5.3 cm, *P* = 0.1301; Fig. 4d). Tumor metabolic activity as measured by total SUVmax was significantly higher in GA ≥ 1 versus GA = 0 cohorts in the fresh blood specimens (48.1 ± 29.5 vs 14.4 ± 12.6 g/ml, *P* = 0.0006; Fig. 4e) and all samples (48.6 ± 30.1 vs 16.9 ± 15.8 g/mL, *P* = 0.0006; Fig. 4f).

## Discussion

Liquid biopsy using plasma cfDNA has been increasingly used as a minimally invasive alternative for tumor genomic profiling [47]. FoundationACT has been recently validated as a clinical NGS assay for genomic profiling of ctDNA derived from cfDNA in blood profiling [29, 30]. As ctDNA only comprises a small fraction of the total cfDNA, sensitive techniques are required to detect sequence alterations in ctDNA that frequently exist at low abundance. Similar to the recent reports using FoundationACT [11], we found that FoundationACT (plasma) assay detected GAs in the majority (75–77%) of our cancer patients (Table 3). While many current efforts have been focused on the technology advances in increasing the sensitivity of plasma ctDNA assays [22], we used the bioinformatics tool MSAF to differentiate ctDNA from cfDNA. The sensitivity of detecting GAs was increased from 77% to 85% in samples with MSAF > 0. The association of detecting GAs in samples with high MSAF and/or high tumor metabolic burden is intuitive. Patients with high tumor metabolic burden produced higher amounts of ctDNA, i.e., higher MSAF numbers (Fig. 4). Similarly, the significant association of detecting GAs with tumor metabolic burden likely reflects more metabolically active tumors shedding larger amounts of ctDNA in blood. These data support our hypothesis that sufficient plasma ctDNA shed from metabolically active tumors is required for the successful detection of GAs in plasma ctDNA. Further study is needed to define the threshold of SUVmax as a screening indicator for each ctDNA assay in histological and molecular homogenous subsets of cancer patients. Moreover, MSAF is an effective bioinformatics tool for identifying the tumor fraction of cfDNA, which can contain a variable amount of germline or other non-tumor DNA (Table 5 and Fig. 3). Ongoing effort aims to define the cutoff of MSAF and further improve the clinical utility of MSAF in quantifying tumor-specific DNA in the plasma. To the best of our knowledge, this is the first study that has systemically and quantitatively evaluated the impact of cfDNA, ctDNA (as MSAF), tumor anatomic burden, and tumor metabolic burden on the ability to detect clinically actionable GAs using a clinical NGS assay for plasma ctDNA. In addition to our primary findings, FoundationACT also detected resistant mutations in two patients, one each with advanced NSCLC and breast cancer, which were not present on the tissue-based FoundationOne assay. This may reflect tumor heterogeneity and/or acquired resistant alteration following treatment, which could have important implications on future treatment selection.

Our study has several limitations. First, we included cancer patients with multiple cancer types at initial diagnosis and tumor progression who subsequently underwent different treatment regimens. The majority (73%) of cases in this study was NSCLC. Further study should consider the impact of different molecular subtypes and cancer types on tumor metabolic activity that might affect the production of plasma ctDNA. Secondly, current clinical reports only use SUVmax as the PET biomarker and do not routinely measure SUVmax for all metabolically active tumor lesions, particularly in patients with extensive metastatic disease, which can significantly alter the value of the metabolic tumor sum. Conversely, our quantification of tumor metabolic burden was labor-intensive and subjected to observer variations. Further investigation should explore automated imaging tools to allow fast and objective quantification of the SUVmax sum and define the detection threshold in each cancer type for each assay for broad clinical application. Thirdly, FDG PET could not distinguish increased metabolism caused by tumor cells from that caused by infectious or noninfectious inflammation such as pneumonitis. Development of tumor-specific molecular imaging might improve the distinction of these clinical entities.

## Conclusion

This study supports using tumor genomic profiling assay for detecting GAs in plasma ctDNA of cancer patients with metabolically active tumors. This knowledge is important for clinicians to select the appropriate patients for tumor genomic profiling assay using plasma ctDNA. Further studies are needed to optimize the clinical application of plasma ctDNA NGS assays in cancer types other than NSCLC.

## Abbreviations

AF: Allele frequency
CAP: College of American Pathologists
cfDNA: Cell-free DNA
CLIA: Clinical Laboratory Improvement Amendments
CR: Complete response
ctDNA: Circulating tumor DNA
dbSNP: Single Nucleotide Polymorphism Database
ExAC: Exome Aggregation Consortium
FDG: Fluorodeoxyglucose
GAs: Genomic alterations
LUAD: Lung adenocarcinoma
LUSC: Lung squamous cell carcinoma
MAF: Mutation allele frequency
MSAF: Maximum somatic allele frequency
NA: Not available
NCCN: National Comprehensive Cancer Network
NGS: Next-generation sequencing
NSCLC: Non-small-cell lung cancer
PD: Progressive disease
PET/CT: Positron emission tomography with computed tomography
PPV: Positive predictive value
PR: Partial response
RECIST: Response Evaluation Criteria in Solid Tumors
SD: Stable disease
SD: Standard deviation
SUVmax: Maximum standardized uptake value
VUS: Variant of unknown significance
